# Fixed-cycle oral pomalidomide/lenalidomide-based therapy is associated with durable treatment-free remission in idiopathic multicentric castleman disease with TAFRO syndrome: a single-center retrospective study

**DOI:** 10.1186/s13023-026-04516-x

**Published:** 2026-08-08

**Authors:** Tang Bo, Xie Shuanglian, Yu Xiaojuan, Wang Wei, Shen Ye, Di Mingyi, Sun Yuhua, Liu Wei, Dong Yujun

**Affiliations:** 1 Department of Hematology, Peking University First Hospital, Beijing, 100034 China; 2 Department of Nephrology, Peking University First Hospital, Beijing, 100034 China; 3 Department of Pathology, Peking University First Hospital, Beijing, 100034 China

**Keywords:** Idiopathic multicentric castleman disease, TAFRO syndrome, Pomalidomide, Lenalidomide, Immunomodulatory drugs, Fixed-cycle therapy, Treatment-free remission

## Abstract

**Background:**

Idiopathic multicentric Castleman disease with TAFRO syndrome (iMCD-TAFRO) is a rare and severe subtype of iMCD characterized by thrombocytopenia, anasarca, fever, reticulin fibrosis, and organomegaly. Current treatments often require prolonged or indefinite administration and frequent hospital visits, imposing substantial burdens on patients. We evaluated the clinical outcomes and safety of a fixed-cycle oral immunomodulatory drug (IMiD)-based regimen in patients with iMCD-TAFRO.

**Methods:**

In this single-center retrospective study, seven patients with iMCD-TAFRO who received an oral immunomodulatory drug (IMiD)-based fixed-cycle regimen were analyzed. Six patients received pomalidomide-dexamethasone or lenalidomide-dexamethasone without concurrent intravenous therapy, whereas one patient received pomalidomide-dexamethasone plus concurrent siltuximab as the initial treatment strategy. The primary endpoint was the overall response rate (ORR) at the prespecified 8-week (± 2 weeks) assessment window. Key secondary endpoints included paired changes in C-reactive protein (CRP), albumin, platelet count, and serum creatinine (Scr) from baseline to month 2. Best overall response was analyzed as an exploratory endpoint.

**Results:**

At the 8-week assessment, four of seven patients achieved a partial response (ORR, 57.1%; 95% CI, 18.4–90.1%), while three had stable disease; no complete responses were observed at this time point. With continued therapy and follow-up, all seven patients achieved complete response as their best overall response (best ORR, 100%; 95% CI, 59.0-100.0%). The median time to initial response was 2.0 months (IQR, 2.0-3.5). Paired analyses from baseline to month 2 demonstrated statistically significant changes in CRP, albumin, and serum creatinine, whereas platelet counts showed numerical improvement without reaching statistical significance. The median treatment duration was 6 months (range, 2–12). Six patients remained in treatment-free remission at last follow-up, with a median treatment-free remission duration of 15.5 months (range, 4–39). One patient relapsed after a prolonged TTNT of 84 months from treatment initiation. Grade 3 adverse events were limited to hyperbilirubinemia (*n* = 1) and febrile neutropenia (*n* = 1); no thromboembolic events or secondary malignancies occurred.

**Conclusions:**

This fixed-cycle oral IMiD-based regimen, with or without concurrent anti-IL-6 therapy in selected cases, was associated with encouraging clinical and laboratory responses in patients with iMCD-TAFRO, including improvements in inflammatory markers, vascular leak, and renal function. Durable treatment-free remission after a finite course of therapy was observed in most patients in this cohort. Given the retrospective design and limited sample size, these findings should be interpreted cautiously and require confirmation in larger multicenter prospective studies.

**Supplementary Information:**

The online version contains supplementary material available at https://doi.org/10.1186/s13023-026-04516-x.

## Background

Idiopathic multicentric Castleman disease (iMCD) is a rare lymphoproliferative disorder characterized by systemic inflammation and multicentric lymphadenopathy [1]. The TAFRO subtype (iMCD-TAFRO) is distinguished by a constellation of severe features—thrombocytopenia, anasarca, fever, bone marrow reticulin fibrosis, and organomegaly—and carries a substantially worse prognosis than non-TAFRO subtypes [2, 3]. The pathogenesis of iMCD-TAFRO involves dysregulated cytokine production, particularly of interleukin-6 (IL-6), and an abnormal immunological milieu that drives systemic inflammation and progressive organ dysfunction [4, 5].

Current first-line therapy for iMCD-TAFRO consists of anti-IL-6 pathway agents (siltuximab or tocilizumab) [6]. However, a substantial proportion of patients experience primary non-response or disease relapses [3, 7]. Combination regimens incorporating rituximab with chemotherapy or immunomodulatory agents have been explored, yet chemotherapy-related toxicity limits long-term use [8]. Thalidomide, a first-generation immunomodulatory drug, has shown clinical activity in small case series, but its requirement for prolonged administration and significant adverse effects—including peripheral neuropathy and teratogenicity—restrict its clinical applicability [9].

International evidence-based guidelines from the Castleman Disease Collaborative Network (CDCN) consider iMCD an incurable disease and recommend indefinite maintenance therapy for responding patients [10]. However, because iMCD-TAFRO is characterized by a cytokine storm–like pathophysiology, there is growing debate about whether treatment can be safely discontinued once the acute inflammatory phase resolves, rather than maintained indefinitely [11–13]. Existing data suggest that sustained treatment-free remission following discontinuation remains uncommon: a systematic review of tocilizumab in TAFRO syndrome (*n* = 31) reported a complete response (CR) rate of only 51.6%, with merely two patients maintaining treatment-free remission at last follow-up [14]. These observations highlight the need to explore treatment strategies that may achieve deep and durable responses within a finite treatment course.

Against this backdrop, lenalidomide and pomalidomide, second-generation immunomodulatory drugs (IMiDs), have emerged as attractive candidates for treating iMCD-TAFRO. These agents exert multifaceted effects encompassing immune modulation, anti-angiogenic activity, and inhibition of abnormal cell proliferation [15, 16]. Importantly, an oral IMiD-based fixed-cycle regimen could offer practical advantages, including improved compliance, outpatient feasibility, and reduced treatment burden, qualities that are particularly relevant for this patient population.

In this retrospective single-center study, we describe the clinical outcomes of seven patients with iMCD-TAFRO who received a fixed-cycle oral pomalidomide- or lenalidomide-based regimen, including one patient who received concurrent siltuximab as part of the initial treatment strategy. Our objective was to evaluate the clinical outcomes, safety, and feasibility of this treatment approach.

## Methods

### Study design and participants

This single-center retrospective study was conducted at Peking University First Hospital and approved by the institutional Ethics Committee (approval number: 2021YAN323). Given its retrospective nature, the requirement for written informed consent was waived. Patients diagnosed with iMCD-TAFRO who received the study regimen for at least 2 months between May 2017 and March 2025 were included.

Patients were excluded if they did not meet the CDCN diagnostic criteria for iMCD-TAFRO; had alternative diagnoses or mimicking conditions, including HHV-8-associated multicentric Castleman disease, POEMS syndrome, lymphoma, active infection, autoimmune disease, or malignancy explaining the clinical presentation; received the IMiD-based regimen for less than 2 months and therefore could not be evaluated at the prespecified 8-week assessment window; or had insufficient clinical or laboratory follow-up data for response assessment.

The diagnosis of iMCD adhered to the CDCN consensus criteria [17], and the TAFRO subtype was defined by the presence of all of the following:

### Histopathological criteria (all required)

- Characteristic lymph node pathology, including atrophic germinal centers with enlarged endothelial cell nuclei, proliferation of endothelial venules, and small numbers of mature plasma cells.
- Negative LANA-1 staining (excluding HHV-8 infection).

### Major clinical criteria (≥ 3 of 5 required)

- Thrombocytopenia (platelet count < 100 × 10⁹/L).
- Anasarca (pleural effusion and/or ascites documented by computed tomography).
- Fever (body temperature > 38 °C).
- Reticulin fibrosis (on bone marrow biopsy).
- Organomegaly (hepatomegaly and/or splenomegaly).

### Minor criteria (≥ 1 required)

- Megakaryocytic hyperplasia or normoplasia in bone marrow.
- Elevated alkaline phosphatase without markedly elevated transaminases.

### Treatment regimen

All patients received an IMiD-based regimen plus dexamethasone, selected by the treating physician in consultation with the patient based on clinical status and tolerability:

1. **PD regimen**: Pomalidomide 4 mg orally on days 1–21 of a 28-day cycle, plus dexamethasone 20 mg orally on days 1–2, 8–9, 15–16, and 22–23.
2. **RD regimen**: Lenalidomide 25 mg orally on days 1–21 of a 28-day cycle, plus dexamethasone 20 mg orally on days 1–2, 8–9, 15–16, and 22–23.

Dexamethasone was administered at the planned dose regardless of baseline renal insufficiency, and no renal function-based dose reduction was performed in this cohort. Renal function, fluid status, infection, glycemic control, and other steroid-related adverse events were monitored during treatment.

Patient 5 received pomalidomide-dexamethasone plus concurrent siltuximab as the initial treatment strategy. Siltuximab was administered once every 21 days for a total of 12 cycles. This combination was selected after shared decision-making because of severe active disease and the patient’s preference to maximize the likelihood and speed of disease control.

Treatment continued until disease progression, unacceptable toxicity, achievement of sustained CR, or patient/physician decision to discontinue. Sustained CR was defined as complete response confirmed at two consecutive clinical assessments at least 4 weeks apart, with no evidence of recurrent systemic symptoms, biochemical inflammation, or radiological progression. Antithrombotic prophylaxis with low-dose aspirin was administered when platelet counts were ≥ 50 × 10⁹/L. Patients intolerant to pomalidomide were switched to lenalidomide at the discretion of the treating physician.

### Safety assessment

Adverse events (AEs) were graded using the National Cancer Institute Common Terminology Criteria for Adverse Events (NCI-CTCAE), version 5.0. All AEs of grade 1 or higher were documented during each treatment cycle. Safety assessments extended from treatment initiation until 30 days after the last dose.

### Response evaluation and endpoints

Treatment response was assessed according to the CDCN consensus response criteria, integrating symptomatic, biochemical, and radiological components [10]. The primary endpoint was the overall response rate (ORR), defined as the proportion of patients achieving CR or partial response (PR) within the prespecified 8-week assessment window (week 8 ± 2 weeks). Key secondary endpoints included the CR rate at week 8 and paired changes in CRP, albumin, platelet count, and Scr from baseline to month 2. Exploratory endpoints included: (i) best overall response (BOR), defined as the best response documented from treatment initiation through follow-up, including responses observed after treatment discontinuation; (ii) time to response (TTR), defined as the interval from treatment initiation to first documented CR or PR; (iii) time to next treatment (TTNT), defined as the interval from regimen initiation to subsequent systemic therapy, death, or last follow-up; and (iv) biomarker changes at BOR. This BOR definition was used because delayed response maturation after finite treatment was a specific clinical feature of interest in this study.

Exposure strata were predefined as: (1) oral pomalidomide-based therapy without concurrent intravenous agents; (2) oral lenalidomide-based therapy without concurrent intravenous agents; and (3) IMiD-based therapy plus concurrent intravenous anti-IL-6 therapy. A sensitivity analysis excluding Patient 5, who received concurrent siltuximab, was performed.

### Follow-up

All patients were actively followed until data cutoff (January 1, 2026). Survival status and subsequent treatments were ascertained through outpatient visits, structured telephone contacts, and review of electronic medical records.

### Statistical analysis

Given the small sample size and non-normal data distributions, paired comparisons between baseline and follow-up values were performed using the Wilcoxon signed-rank test. Effect sizes were summarized as Hodges-Lehmann (HL) median differences with 95% confidence intervals (CIs) derived from Walsh averages. ORR was reported with exact Clopper-Pearson 95% CIs. TTR was summarized as median with interquartile range (IQR). Treatment-free remission duration was summarized among patients who remained in remission at last follow-up, whereas TTNT was reported separately for the patient who relapsed. Follow-up laboratory values were defined as those at the earliest CR time point (if achieved) or at the PR time point. All analyses were performed using SPSS 29.0.

## Results

### Patient characteristics

Seven patients (3 males, 4 females) were included, with a median age at treatment initiation of 44 years (range, 30–59). Histopathological analysis revealed plasmacytic features in three patients (42.9%) and mixed features in four (57.1%); none exhibited the hyaline vascular subtype. Representative diagnostic lymph-node and renal-biopsy findings from Patient 6 are shown in Fig. 2. All patients had an Eastern Cooperative Oncology Group (ECOG) performance status of ≥ 2, reflecting the severity of disease at presentation.

The most prevalent clinical manifestations at baseline were fatigue (7/7, 100%), serous effusions (7/7, 100%), weight change (7/7, 100%), fever (5/7, 71.4%), and splenomegaly (5/7, 71.4%). No patients had cutaneous or pulmonary involvement. Baseline laboratory values were markedly abnormal: the median hemoglobin was 76 g/L (range, 59–97), platelet count 62 × 10⁹/L (range, 8–349), erythrocyte sedimentation rate (ESR) 58 mm/h (range, 15–122), CRP 107.1 mg/L (range, 4.9–236.2), albumin 27.2 g/L (range, 22.0–31.2), Scr 242 µmol/L (range, 151–320), and IL-6 14.22 pg/mL (range, 4.6–94.16). Complete baseline characteristics are presented in Table 1.

**Table 1:** Baseline characteristics of patients with iMCD-TAFRO (*n* = 7)

Characteristic	Reference range	Value
Age, median (range), years	—	44 (30–59)
Male sex, n (%)	—	3 (42.9)
**Histological subtype**,** n (%)**		
Hyaline vascular	—	0 (0)
Plasmacytic	—	3 (42.9)
Mixed	—	4 (57.1)
**ECOG performance status**,** n (%)**		
< 2	—	0 (0)
≥ 2	—	7 (100)
**Clinical features**,** n (%)**		
Fever	—	5 (71.4)
Weight change	—	7 (100)
Peripheral lymphadenopathy	—	7 (100)
Splenomegaly	—	5 (71.4)
Skin involvement	—	0 (0)
Pulmonary involvement	—	0 (0)
Anasarca	—	7 (100)
**Laboratory values**,** median (range)**		
IL-6, pg/mL	< 6.4	14.22 (4.6–94.16)
Hemoglobin, g/L	115–175	76 (59–97)
Platelet count, × 10⁹/L	125–350	62 (8–349)
Serum creatinine, µmol/L	44–133	242 (151–320)
CRP, mg/L	0–3	107.1 (4.9–236.2)
ESR, mm/h	0–15	58 (15–122)
Albumin, g/L	40–55	27.2 (22.0–31.2)
IgG, g/L	7–16	10.8 (5.5–14.0)

Abbreviations: CRP, C-reactive protein; ECOG, Eastern Cooperative Oncology Group; ESR, erythrocyte sedimentation rate; IgG, immunoglobulin G; IL-6, interleukin-6

### Treatment outcomes

At the prespecified 8-week (± 2 weeks) assessment, four of seven patients achieved PR (ORR, 57.1%; 95% CI, 18.4–90.1%), while three had stable disease (SD); no CR was observed at this time point. With continued therapy and follow-up, all seven patients achieved CR as their best overall response (BOR ORR, 100%; 95% CI, 59.0-100.0%). The median TTR was 2.0 months (IQR, 2.0-3.5). Notably, three patients with SD at week 8 subsequently deepened their responses, consistent with delayed response maturation.

The median treatment duration was 6 months (range, 2–12 months). Six patients remained in treatment-free remission at last follow-up, with a median treatment-free remission duration of 15.5 months (range, 4–39). One patient (Patient 7) relapsed after a prolonged TTNT of 84 months from treatment initiation.

Patient-level treatment timelines and outcomes are summarized in Table 2. Patient 5 received PD plus concurrent siltuximab as the initial treatment strategy, achieved CR by month 4, completed 12 months of therapy, and remained in remission for 9 months after treatment discontinuation. Patient 6 had SD at month 2 and PR at month 3; treatment was discontinued at month 6 with sustained PR, and CR was documented 2 months after cessation, with ongoing remission during follow-up. Patient 7 received RD without concurrent siltuximab, had SD at month 2, achieved CR at month 6, and relapsed at 84 months after treatment initiation.

**Table 2:** Patient-level treatment timeline and outcomes

Patient	Regimen	Start date	Week-8 response	Best overall response	TTR (months)	Treatment duration (months)	Treatment-free remission duration / TTNT (months)	Current status
1	PD	Aug 2022	PR	CR	2	2	39	Remission
2	PD	Sep 2022	PR	CR	2	4	36	Remission
3	PD	Mar 2024	PR	CR	2	6	16	Remission
4	PD→RD	Aug 2024	PR	CR	2	6	15	Remission
5	PD + siltuximab	Apr 2024	SD	CR	4	12	9	Remission
6	PD	Mar 2025	SD	CR	3	6	4	Remission
7	RD	May 2017	SD	CR	6	6	84	Relapsed

Abbreviations: CR, complete response; PD, pomalidomide-dexamethasone; PR, partial response; RD, lenalidomide-dexamethasone; SD, stable disease; TTR, time to response; TTNT, time to next treatment. The final column denotes treatment-free remission duration for patients remaining in remission; for Patient 7, 84 months denotes TTNT from treatment initiation. Patient 5 received concurrent siltuximab as part of the initial treatment strategy and was excluded from the sensitivity analysis of patients treated without concurrent intravenous therapy

In the prespecified sensitivity analysis excluding Patient 5, who received concurrent intravenous siltuximab, the best ORR remained 100% (6/6; 95% CI, 54.1–100.0%), with all six patients achieving CR as their best overall response. At week 8, four of six patients in this subgroup achieved PR (ORR, 66.7%; 95% CI, 22.3–95.7%).

### Laboratory outcomes

**Fig. 1:**
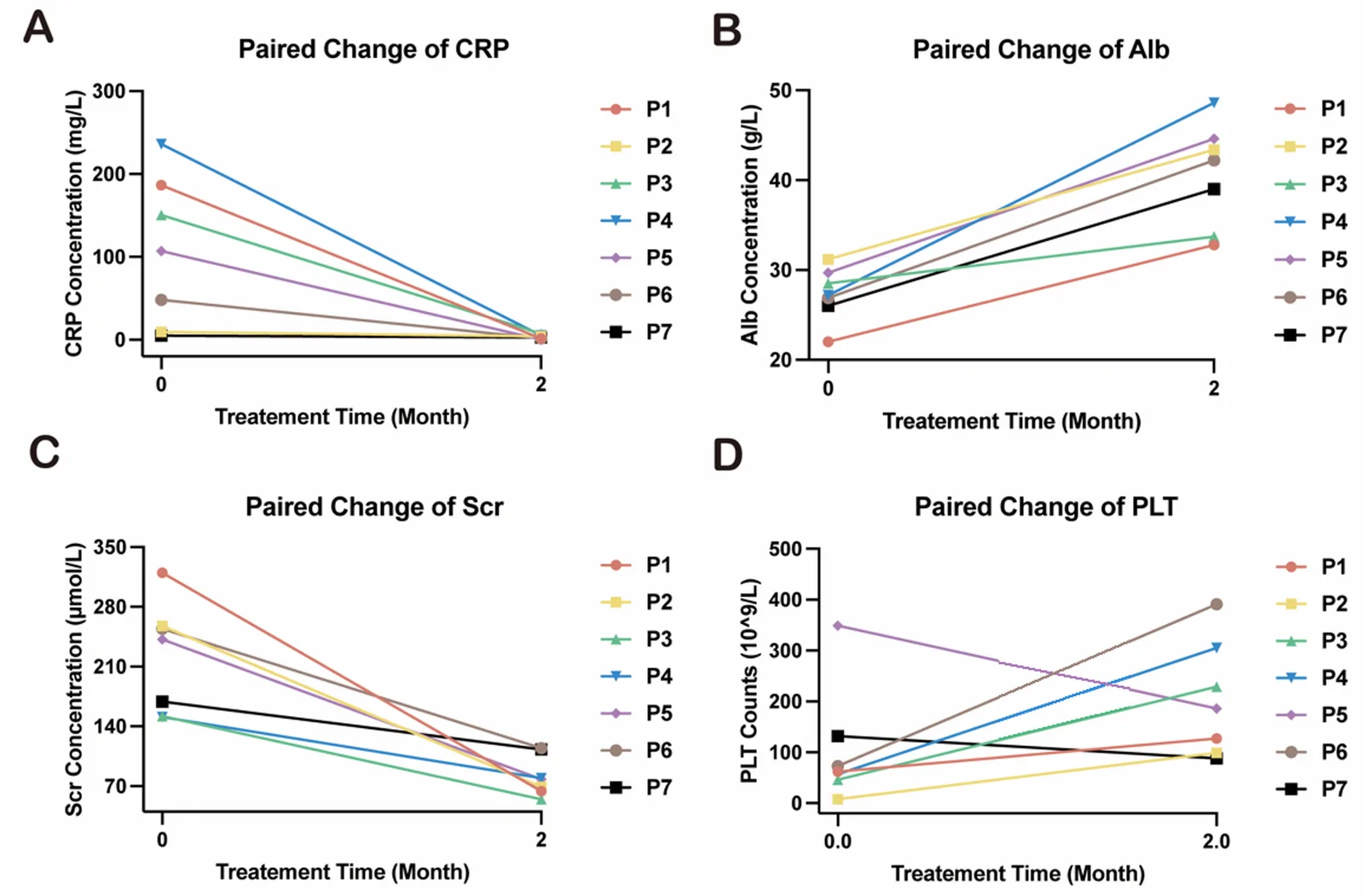
Paired changes in laboratory parameters from baseline to 2 months after treatment initiation. Each line represents an individual patient. (**A**) C-reactive protein (CRP), (**B**) albumin, (**C**) serum creatinine, and (**D**) platelet count

Paired laboratory analyses at month 2 demonstrated early improvement in markers of systemic inflammation, vascular permeability, and renal function (Fig. 1). Median CRP decreased from 107.09 mg/L (IQR, 28.86–168.51) at baseline to 2.90 mg/L (IQR, 1.55–4.71) at month 2 (HL Δ, − 101.07; 95% CI, − 187.01 to − 24.32; *p* = 0.016). Median albumin increased from 27.2 g/L (IQR, 26.45–29.10) to 42.2 g/L (IQR, 36.35–44.00; HL Δ, + 13.18; 95% CI, 9.10–18.15; *p* = 0.016). Median Scr decreased from 242.0 µmol/L (IQR, 160.55–256.05) to 78.11 µmol/L (IQR, 66.55–96.20; HL Δ, − 135.31; 95% CI, − 197.90 to − 76.77; *p* = 0.016). Platelet counts increased numerically from 62.0 × 10⁹/L (IQR, 51.5–102.5) to 186.0 × 10⁹/L (IQR, 113.0–267.0), but this change did not reach statistical significance (HL Δ, + 96.5; 95% CI, − 49.0 to 250.5; *p* = 0.156).

Laboratory changes at best overall response are detailed in the Supplementary Materials (Supplementary Table S1; Supplementary Fig. S1).

**Fig. 2:**
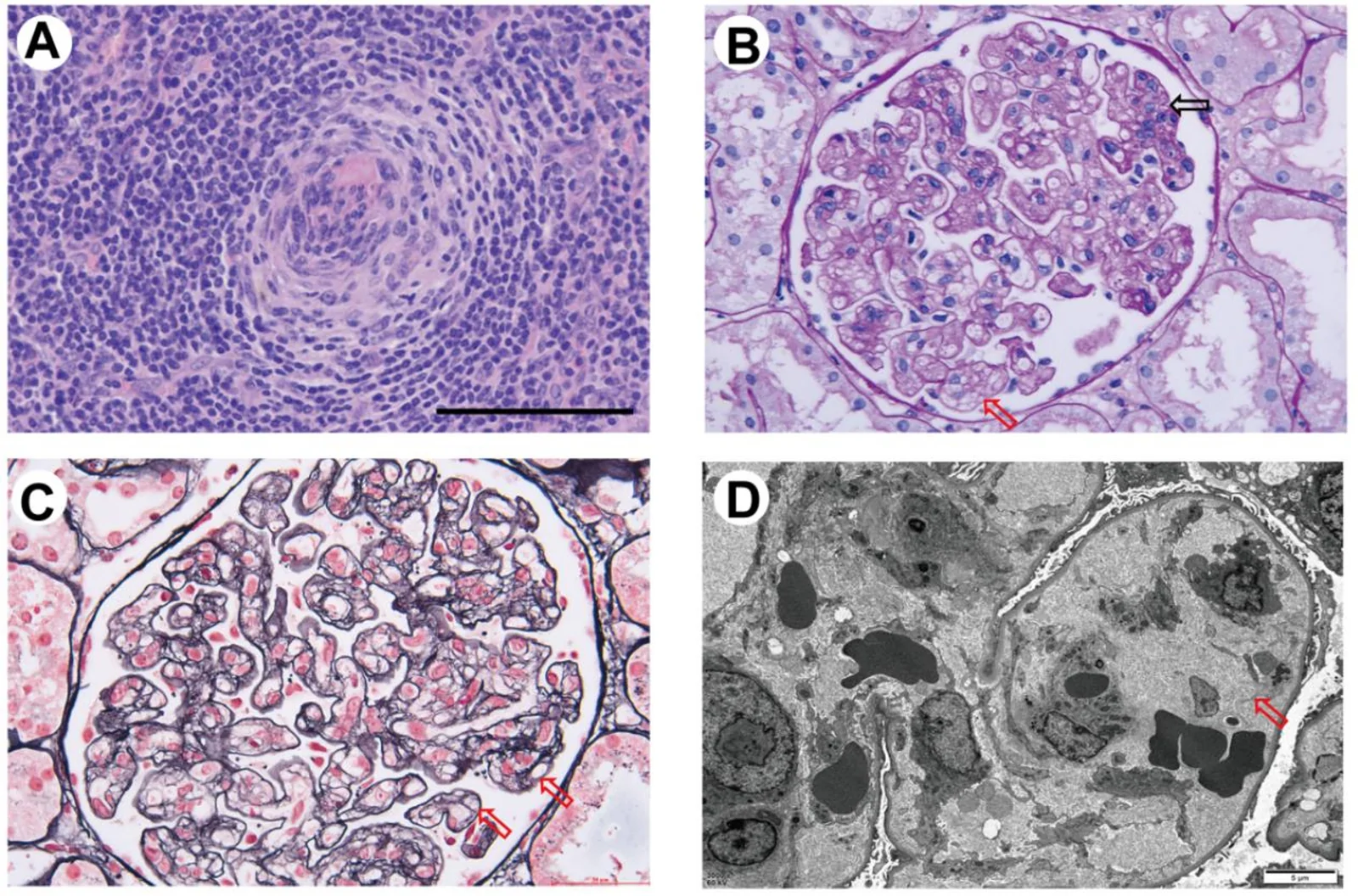
Representative histopathological and ultrastructural findings in Patient 6. The lymph node and renal biopsy specimens were obtained from Patient 6 during the diagnostic work-up before initiation of IMiD-based therapy. (**A**) Lymph node biopsy, hematoxylin and eosin staining, ×400, showing Castleman disease-like lymph node changes with atrophic germinal centers, concentric lymphocytic mantle zones, and prominent hyalinized vessels penetrating the follicle. Scale bar = 100 μm. (**B**) Kidney biopsy, periodic acid-Schiff staining, ×400, showing glomerular microangiopathy with endothelial cell swelling and proliferation, and subendothelial edema. Scale bar = 50 μm. (**C**) Kidney biopsy, periodic acid-methenamine silver plus Masson trichrome staining, ×400, showing segmental double-contour formation of the glomerular capillary wall and mild mesangial matrix expansion, consistent with chronic endothelial injury. Scale bar = 50 μm. (**D**) Electron microscopy showing diffuse widening of the lamina rara interna of the glomerular basement membrane, extensive podocyte foot process effacement, and endothelial cell swelling, without electron-dense immune deposits. Scale bar = 5 μm

### Safety

No treatment-related deaths occurred. The most common grade 1–2 AEs were fatigue (5/7, 71.4%), rash (1/7, 14.3%), and constipation (1/7, 14.3%), all of which were managed with supportive care. Grade 3 AEs occurred in two patients: one case of hyperbilirubinemia (which resolved after treatment discontinuation) and one case of febrile neutropenia (which resolved with supportive care and prompted a switch from PD to RD). No thromboembolic events or secondary malignancies were observed during the study period.

## Discussion

In this single-center retrospective study of seven patients with iMCD-TAFRO, a fixed-cycle oral IMiD-based regimen was associated with encouraging clinical activity and a favorable safety profile. All patients achieved complete response as their best overall response during follow-up, and most remained in treatment-free remission after a finite course of therapy. These findings suggest that a time-limited, outpatient-based treatment strategy may be feasible for selected patients with iMCD-TAFRO, although confirmation in larger prospective studies is required.

iMCD-TAFRO is the most clinically aggressive subtype of iMCD, with a 3-year overall survival rate of only 65.7% in a Chinese nationwide multicenter study, substantially inferior to the 87–94% reported for iMCD-NOS [18]. Most patients present with severe, life-threatening disease at onset, and the risk of mortality is concentrated in the early decompensation phase [19, 20]. Anti-IL-6 pathway agents (siltuximab and tocilizumab) constitute the current first-line therapy [10], yet their efficacy varies considerably: the ORR of siltuximab was 34% in the pivotal randomized trial [21] and 52–71% in real-world studies [22, 23]. A significant proportion of patients experience primary non-response or secondary failure, necessitating escalation to cytotoxic chemotherapy, which carries substantial toxicity and limited long-term tolerability [24]. Alternative therapeutic strategies have been explored for refractory iMCD, including targeting PI3K/AKT/mTOR signaling, Janus kinase inhibition, and other immunomodulatory approaches [25–27]. Predictive models and combination regimens incorporating rituximab and bortezomib have also been investigated to support treatment selection and improve outcomes in iMCD [28, 29]. Emerging oral options are also being explored. Orelabrutinib, a Bruton’s tyrosine kinase inhibitor, has shown activity in relapsed/refractory iMCD, and delayed deepening of response has also been observed with this oral approach [30]. These data support the broader need for practical oral strategies, while underscoring that evidence for each approach remains limited.

A critical unresolved question in iMCD management is whether and when treatment can be safely discontinued. CDCN guidelines recommend indefinite maintenance therapy [10], yet this approach imposes a considerable burden on patients and healthcare systems. In practice, long-term treatment-free remission has been rarely documented: in a systematic review of tocilizumab for TAFRO syndrome, only 2 of 31 patients maintained treatment-free remission at last follow-up [14]. These observations highlight the need to further investigate treatment strategies that may achieve deep and durable responses within a defined treatment period.

The ORR of 57.1% at the prespecified 8-week assessment and the best ORR of 100% observed during follow-up suggest that treatment with this regimen was associated with clinically meaningful responses that deepened over time. The median TTR of 2 months suggests that clinical responses were observed relatively early after treatment initiation, with significant improvements in CRP, albumin, and Scr observed within the first 2 months. The magnitude of CRP reduction (HL Δ, − 101.07 mg/L) and albumin recovery (HL Δ, + 13.18 g/L) within this timeframe suggests concurrent suppression of systemic inflammation and reversal of capillary leak, both of which are central to TAFRO pathophysiology. The substantial improvement in Scr (HL Δ, − 135.31 µmol/L) further suggests that renal dysfunction—a major contributor to morbidity in iMCD-TAFRO—may be at least partially reversible with this approach. Of note, the non-significant change in platelet count at month 2 may reflect the slower kinetics of hematopoietic recovery relative to inflammation resolution, although all patients demonstrated numerical improvement.

The pattern of delayed response maturation—with three patients having SD at week 8 but subsequently achieving CR—has clinical implications for the timing of response assessment. Premature discontinuation based on an early assessment may deny patients the opportunity for deeper responses, suggesting that continued therapy beyond the initial 2 months may be considered in patients without evidence of disease progression.

A distinctive feature of this regimen is its finite treatment duration. The median treatment course of 6 months (range, 2–12) contrasts with the indefinite maintenance required by anti-IL-6 agents and with the longer treatment exposure reported for the thalidomide-cyclophosphamide-prednisone (TCP) regimen. In the phase 2 TCP study by Zhang et al., the median treatment duration was approximately 13 cycles, and adverse events including peripheral neuropathy were observed [9]. In comparison, the present IMiD-based regimen was associated with a median treatment duration of 6 months and manageable toxicity in this small cohort. Cross-study comparisons should be interpreted cautiously because of differences in study design, patient population, disease severity, and sample size. Six of seven patients remained durable treatment-free remission after completing therapy, with a median treatment-free remission duration of 15.5 months (range, 4–39). Although Patient 7 relapsed after a TTNT of 84 months from treatment initiation, the prolonged TTNT suggests that even in cases of eventual relapse, the treatment-free interval can be clinically meaningful.

These observations are consistent with the hypothesis that iMCD-TAFRO, as a cytokine storm–driven disease, may be amenable to treatment discontinuation once the inflammatory cascade is effectively interrupted [11, 12]. However, given the small sample size, the identification of reliable biomarkers for predicting safe discontinuation remains an important objective for future research.

The pleiotropic mechanisms of IMiDs may underlie the multidimensional efficacy observed in this study. Pomalidomide and lenalidomide bind cereblon (CRBN) and promote the degradation of the lymphoid transcription factors IKZF1 and IKZF3, thereby modulating T-cell and natural killer cell function and directly suppressing monocyte-derived IL-6 and TNF-α secretion [31, 32]. Recent tissue-based work suggests that, in iMCD-TAFRO, IL-6 may be derived predominantly from vascular endothelial cells rather than lymph node plasma cells, contrasting with IPL-type disease [4, 33]. This endothelial-cell-centered biology provides a plausible rationale for therapies with anti-angiogenic and endothelial-stabilizing properties. IMiDs exert anti-angiogenic effects through downregulation of vascular endothelial growth factor (VEGF), which may contribute to stabilization of endothelial barrier integrity and rapid resolution of anasarca, hypoalbuminemia, and renal dysfunction [34]. By suppressing the inflammatory and vascular-leak milieu, these agents may also create conditions favorable for recovery of bone marrow hematopoietic function and renal parenchymal repair [35].

Renal involvement is a clinically important feature of iMCD-TAFRO and may reflect a spectrum of pathological injury, including thrombotic microangiopathy-like endothelial injury, membranoproliferative glomerulonephritis-like changes, and tubulointerstitial damage [35]. The renal biopsy from Patient 6, performed during the diagnostic work-up before treatment, showed glomerular microangiopathy, with endothelial swelling and proliferation, subendothelial edema, double-contour formation of the capillary wall, and widening of the lamina rara interna on electron microscopy, without electron-dense immune deposits (Fig. 2). These findings are consistent with endothelial injury as a key renal pathological feature in TAFRO and are biologically aligned with the vascular-leak phenotype of the disease. The marked improvement in serum creatinine observed during follow-up is consistent with partial recovery of renal dysfunction, although causality cannot be established in this retrospective study. However, given that renal pathology was available from only one patient, these findings should be interpreted descriptively and cannot establish a treatment-specific pathological effect.

One patient received concurrent siltuximab as part of the initial treatment strategy, which may confound attribution of response to the oral IMiD-based regimen alone. Therefore, we performed a sensitivity analysis excluding this patient. The persistence of a 100% best ORR in the remaining six patients supports the robustness of the observed activity signal, although the small sample size precludes definitive conclusions.

The safety profile of this regimen was favorable. Grade 3 AEs occurred in only two patients and were manageable: hyperbilirubinemia resolved upon treatment discontinuation, and febrile neutropenia responded to supportive care. The oral IMiD-based backbone reduced reliance on intravenous treatment for six patients, lowering the burden associated with hospital visits, a particularly relevant advantage for patients with ECOG performance status ≥ 2 who may have difficulty tolerating frequent clinic attendance. The absence of thromboembolic events, despite the known prothrombotic risk associated with IMiDs, may be attributable to the concomitant thrombocytopenia characteristic of iMCD-TAFRO and the routine use of aspirin prophylaxis. Taken together, these findings should be interpreted as preliminary and hypothesis-generating. Given the retrospective design, limited sample size, and absence of a comparator group, confirmation in larger prospective multicenter studies is warranted before this treatment approach can be incorporated into routine clinical practice.

### Limitations

Several important limitations should be acknowledged. First, the retrospective, single-center design and small sample size (*n* = 7) limit the generalizability of these findings and preclude definitive conclusions regarding efficacy. Selection bias cannot be excluded, as only patients who received at least 2 months of therapy were included. Second, the absence of a control group precludes direct comparison with established therapies. Third, Patient 5 received concurrent siltuximab as part of the initial treatment strategy, complicating attribution of treatment effects to the IMiD-based regimen alone, although sensitivity analysis excluding this patient yielded consistent findings. Fourth, follow-up duration and treatment-free remission duration varied substantially across patients, and longer observation is needed to characterize late relapse risk and identify prognostic factors. Fifth, the prespecified 8-week assessment window, while chosen to minimize time-point bias, may not capture the full kinetics of response for all patients; future studies should consider incorporating serial assessments at standardized intervals. Finally, renal pathological data were available from only one patient, and potential biomarkers that could predict treatment response, guide discontinuation decisions, and identify patients at risk for relapse warrant investigation in future prospective studies.

## Conclusions

This retrospective single-center study suggests that a fixed-cycle oral IMiD-based regimen, with or without concurrent anti-IL-6 therapy in selected cases, was associated with favorable clinical and laboratory responses in patients with iMCD-TAFRO. Durable treatment-free remission following a finite course of therapy was observed in most patients, supporting the feasibility of further investigating this treatment strategy. Given the retrospective design, limited sample size, and absence of a comparator group, these findings should be interpreted as preliminary and hypothesis-generating. Larger multicenter prospective studies are needed to confirm the efficacy, safety, and long-term durability of this approach.

## Supplementary Information

Below is the link to the electronic supplementary material.

Supplementary Material 1

## Data Availability

No datasets were generated or analysed during the current study.
