# Supplementary material for: Fixed-cycle oral pomalidomide/lenalidomide-based therapy is associated with durable treatment-free remission in idiopathic multicentric castleman disease with TAFRO syndrome: a single-center retrospective study
Source: Orphanet J Rare Dis. 2026 Aug 8;21:401. doi: 10.1186/s13023-026-04516-x (PMC13741506; doi:10.1186/s13023-026-04516-x)
Supplement: Supplementary file 1 [file 13023_2026_4516_MOESM1_ESM.docx]

**Supplementary Materials**

**Best Overall Response (BOR) Assessment**

**Supplementary Table S1. Paired laboratory changes (Wilcoxon + HL)**

| Analyte (n=7) | Baseline median (IQR) | Follow-up median (IQR) | Change median (IQR) | Hodges–Lehmann Δ (95% CI) | Wilcoxon p |
| --- | --- | --- | --- | --- | --- |
| PLT (×10⁹/L) | 62 (52–102) | 169 (142–222) | 128 (52–172) | 128 (-49 to 228) | 0.2969 |
| CRP (mg/L) | 107.09 (28.86–168.51) | 1 (0.59–1.75) | -106.95 (-167.81–-25.96) | -106.95 (-192.75 to -24.82) | 0.0156 |
| Albumin (g/L) | 27.2 (26.4–29.1) | 42.2 (40.3–43) | 14 (13.6–15.4) | 14 (11.25 to 16.95) | 0.0156 |
| Scr (μmol/L) | 242 (160.55–256.05) | 84.8 (71.95–86.75) | -165.9 (-171.63–-72.92) | -165.9 (-214.28 to -69.32) | 0.0156 |

Supplementary Figure S1. Paired changes in laboratory parameters from baseline to best response after treatment. Each line represents one patient. (A) C-reactive protein (CRP), (B) albumin, (C) serum creatinine, and (D) platelet count.
